# Grassland health assessment based on indicators monitored by UAVs: a case study at a household scale

**DOI:** 10.3389/fpls.2023.1150859

**Published:** 2023-09-20

**Authors:** Yifei Luo, Wenxiang Ji, Wenjun Wu, Yafang Liao, Xinyi Wei, Yudie Yang, Guoqiang Dong, Qingshan Ma, Shuhua Yi, Yi Sun

**Affiliations:** ^1^ Institute of Fragile Eco-environment, School of Geographic Science, Nantong University, Nantong, China; ^2^ State Key Laboratory of Grassland Agro-ecosystems, Key Laboratory of Grassland Livestock Industry Innovation, Ministry of Agriculture and Rural Affairs, College of Pastoral Agriculture Science and Technology, Lanzhou University, Lanzhou, China; ^3^ School of Agriculture and Environment, College of Science, Massey University, Palmerston North, New Zealand; ^4^ Forestry Station of Huangnan Prefecture of Qinghai Province, Tongren, China

**Keywords:** grassland ecosystem, grassland health monitor, biological index, FragMAP, environmental condition

## Abstract

Grassland health assessment (GHA) is a bridge of study and management of grassland ecosystem. However, there is no standardized quantitative indicators and long-term monitor methods for GHA at a large scale, which may hinder theoretical study and practical application of GHA. In this study, along with previous concept and practices (i.e., CVOR, the integrated indexes of condition, vigor, organization and resilience), we proposed an assessment system based on the indicators monitored by unmanned aerial vehicles (UAVs)-UAV*_CVOR_*, and tested the feasibility of UAV*_CVOR_* at typical household pastures on the Qinghai-Tibetan Plateau, China. Our findings show that: (1) the key indicators of GHA could be measured directly or represented by the relative counterpart indicators that monitored by UAVs, (2) there was a significantly linear relationship between CVOR estimated by field- and UAV-based data, and (3) the CVOR decreased along with the increasing grazing intensity nonlinearly, and there are similar tendencies of CVOR that estimated by the two methods. These findings suggest that UAVs is suitable for GHA efficiently and correctly, which will be useful for the protection and sustainable management of grasslands.

## 1 Introduction

Grassland covers about one third of the world’s land area, it plays an irreplaceable role in maintaining ecological functions (e.g., carbon storage and water conservation), suppling forage for livestock and wild animals (O’Mara, 2012; Wang et al., 2019). However, grasslands are degraded or degrading continually because of anthropogenic activities (especially grazing) and climate changes (Wang et al., 2019; Wei et al., 2022a). Therefore, it is urgent to estimate grassland health status accurately and efficiently, and furthermore, adapt reclamation activities to make sure the sustainability of the grassland ecosystem.

Grassland ecosystem is a complex system, and it is necessary to establish an integrated grassland health assessment system (GHA, Xu and Guo, 2015). Generally, GHA patterns could be categorized as qualitative or quantitative in nature (Lepak et al., 2022). Qualitative methods estimate or judge grassland conditions based on systematic observations. To date, the most widely adopted qualitative assessment approach is “Interpreting Indicators of Rangeland Health” (IIRH) (Pyke et al., 2002). It estimates three ecosystem attributes, i.e., soil/site stability, biotic integrity, and hydrologic function, and a suite of qualitative indicators collected by interdisciplinary specialists. Despite it was increasing used in USA and Australia, few researchers have applied the IIRH to assess grassland ecosystems health status across large spatial extents (de Soyza et al., 2000a), and a lack of specialists and comparability resulting in inconsistent standards is the major cause limiting the use of IIRH. Quantitative methods were established on numerical indicators, and scientists are always wrestled with how to characterize and keep track of grassland changes using quantitative monitor (Hou et al., 2004; Lepak et al., 2022). The widely adopted quantitative assessment approach is mainly derived from the concept of ecosystem health, e.g., the CVOR integrated index (i.e., condition, vigor, organization and resilience, hereafter COVR) which is proposed based on the VOR index (Hou et al., 2004). CVOR was widely used to assess GHA of various types of grassland in Inner Mongolia (Wang et al., 2008), Ningxia (Yu et al., 2018), and Xijiang Province (Lu et al., 2017). Usually, the quantitative assessing frameworks are developed by process-based studies (Pyke et al., 2002), however, the available resources are often limited and hard to support the work, except for a small number of grassland ecological sites (Miller, 2008), and only focused on the field-based indicators in majority of previous work, which featured with inefficient and labor-intensive (Whitford et al., 1998; de Soyza et al., 2000b; Pyke et al., 2002).

The need for affordable and effective methods across expansive grassland led to lots of efforts focused on the development of indicators that could be reliably detected (de Soyza et al., 2000a; Ludwig et al., 2007; Sun et al., 2018). To date, however, the widely recognized assessment systems are lack, and how to select effective evaluation indexes and establish appropriate evaluation methods with new tools is still largely unknown (Wang et al., 2018). In the last decade, along with unmanned aerial vehicles (UAVs) was applied in studies of species composition (Sun et al., 2018; Sun et al., 2022), vegetation coverage (Chen et al., 2016) and above-ground biomass (Zhang et al., 2018), new opportunities are emerging to enable novel GHA systems that are suitable for multiple scale and featured with high comparability and efficiency.

The Qinghai-Tibetan Plateau (QTP) is the highest plateau in the world, and is often referred to as the Third pole (Yao et al., 2022). Alpine grassland is the most dominant vegetation type, and grazing is the most extensive management mode on the QTP (Harris, 2010). Reasonable grazing management is regarded as the most effective way to utilize and protect alpine grassland (Sun et al., 2020), and GHA is the first step in practical application. Actually, GHA is gradually recognized as a vital component for reasonable management of grassland ecosystems (Herrick et al., 2006; Miller, 2008). In this study, along with previous concept and practices (mainly the CVOR), we proposed an assessment method based on the indicators collected by unmanned aerial vehicles (UAVs) - UAV*_CVOR_*, and tested the feasibility of UAV*_CVOR_* at the typical household pastures on the QTP. Our specific objectives were to: (1) make sure whether UAV*_CVOR_* is suitable for GHA, especially for alpine meadow, (2) compare the differences of CVOR that were estimated by UAV- and field-based sampling methods, and (3) reveal the GHA changes along with the gradients of grazing intensity. Results of this study could have significant implications on reasonable monitor and management of grassland and socio-ecological sustainable development.

## 2 Materials and methods

### 2.1 Study sites

**Figure 1:**
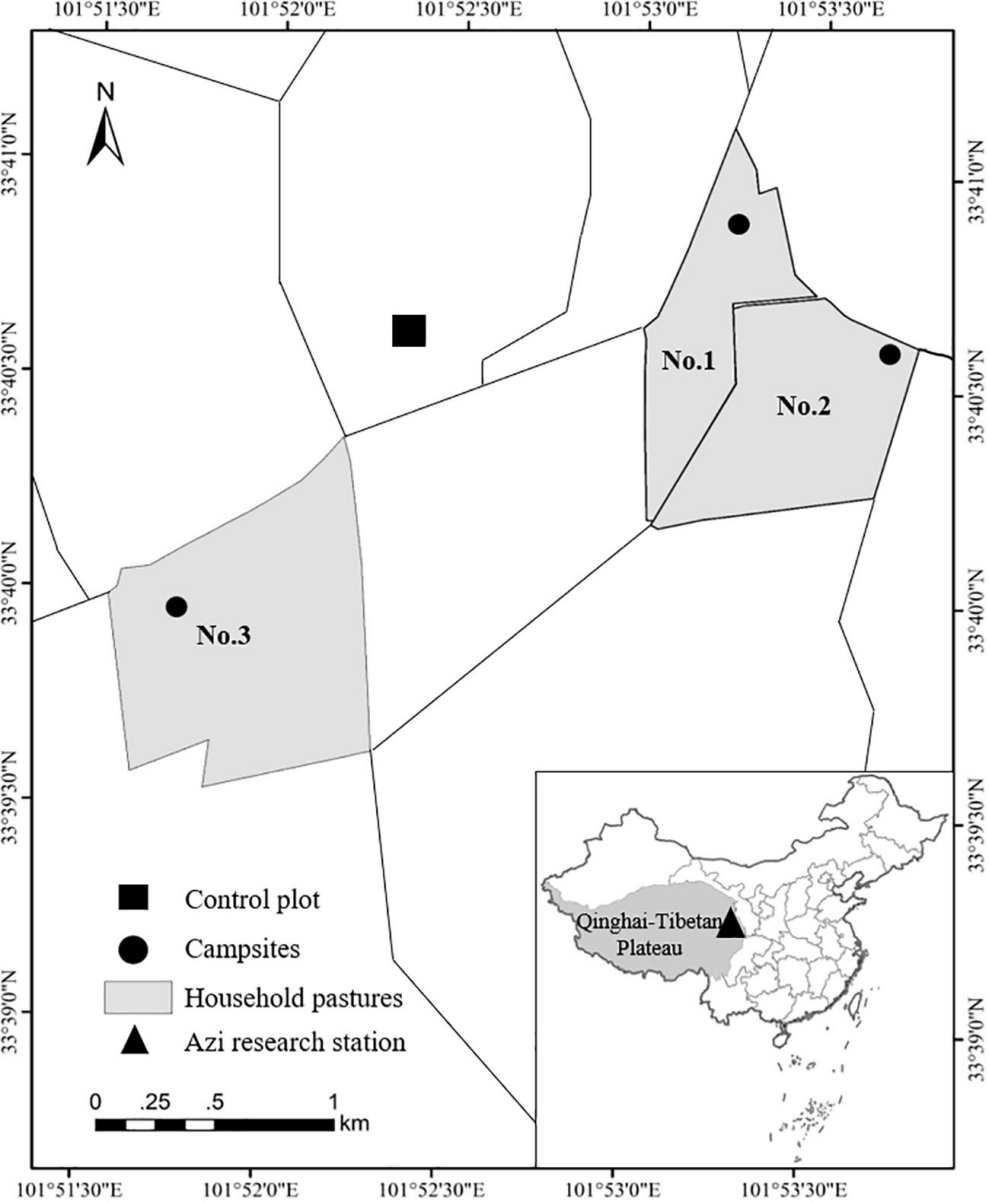
Study area in the Eastern Qinghai-Tibetan Plateau (the triangle in the insert). Gray areas indicate sampling household pastures, lines indicate pasture borders, black circles represent campsites and back square represent the control plot.

In 2017, This study was conducted at Azi Research Station in Gansu Province, China (101°52′07.9″E, 33°24′24.1″N; 3547m a.s.l.) (**Figure 1**). The study area is located in a humid region, mean annual precipitation > 600 mm and mean annual air temperature is approximately 1.1°C (Sun et al., 2015). The soil is alpine meadow soil (Gao and Li, 1995). The plant community is dominated by Poaceae and Cyperaceae, and meanwhile, various dicotyledonous species are also common, e.g., the *Polygonaceae*, *Saxifragaceae*, *Ranunculaceae*, *Asteraceae* (Ma et al., 2010).

Three typical household pastures (the aera is 48.53–113.64 ha) that are primarily used for grazing in warm season were selected, the pastures are gentle topography (slope< 5°) in case the slope and aspect affect the plant and herbivores (**Figure 1**). As conventional grazing pattern, yaks are free grazing during daytime and were penned at night. The pastures we selected have been continually grazed for more than 30 years in this way, and it generates a radial gradient of grazing intensity from the campsites (Chillo et al., 2015; Sun et al., 2018). Therefore, it provides suitable research areas to study the relationships between GHA and grazing intensity, and meanwhile, test the feasibility of the proposed UAV*_CVOR._*

**Figure 2:**
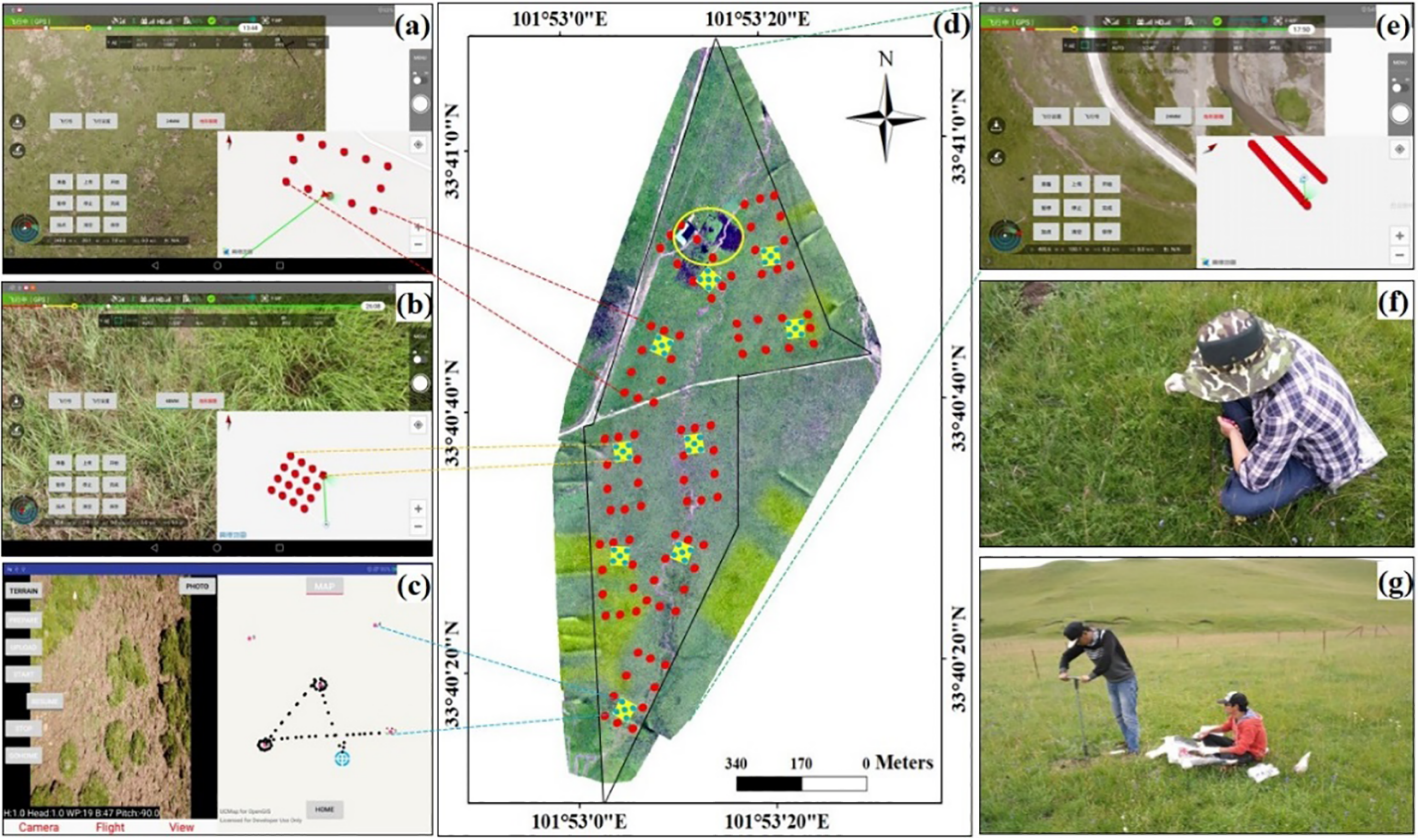
Indicators of grassland health assessment sampling and monitor by unmanned aerial vehicle and field-based verification (Using the No.1 pasture as an example). **(A)** the real-time control interface for *Rectangle* mode, **(B)** *Belt* mode, **(C)** *Quadrat* mode, **(D)** the georeferenced orthomosaic of the studied pasture (dotted black line indicates borders of the pasture, and yellow circle represents campsite area), **(E)** *Mosaic* mode, **(F)** species composition and biomass field-based verification, and **(G)** soil sampling.

Nine sample points (the definition is the centers of *Belt* and *Quadrat* routes in this study, **Figure 2**) were set up randomly between campsites and the margin of the pastures. The distances of the sample points from the corresponding campsite were measured based on georeferenced orthomosaic of the pastures, which were generated using overlapping aerial photographs acquired from the *Mosaic* flight mode (e.g., **Figures 2D, E**). It was recognized that grazing intensities are the proxy parameters of inverse distance from the water point or campsite (Wesuls et al., 2013; Chillo et al., 2015; Sun et al., 2018). Three fencing paddocks (used as control treatments of multi-stocking rates and grazing system experiments) set up since 2010 were selected as the control plots (Sun et al., 2015; Sun et al., 2018). All the monitor missions were conducted with permissions from both the landowner and airspace authority.

### 2.2 Sampling in the field and collecting data

Based on the empirical and experimental studies (Hou et al., 2004; Shan et al., 2012; Yu et al., 2018), the VOR integrated index was used to describe biological characteristics of grassland ecosystems, which included vigor, organization and resilience and were described by aboveground biomass, richness, and the ratio of the species that indicate restoration and degraded degree (RRD) (Shan et al., 2012; Yu et al., 2018). To describe the integrated environmental factor, the soil organic carbon (SOC) was sampled to establish a new practical integrated index - CVOR (Hou et al., 2004). In this study, we followed the previous work and calculated the CVOR using the same methods at each sample point (Hou et al., 2004; Shan et al., 2012; Yu et al., 2018). To explore relationships between the CVOR values that were estimated by field-and UAV-based methods, three quadrats were sampled within the *Quadrat* routes randomly (detailed as below). The aboveground biomass and species composition were measured and average values were used in statistical analyses.

UAV-based sampling was also conducted. At each sample point, a *Belt* (the ground sampling distance was about 0.09 cm and covered 2.6 m × 3.5 m on ground), *Quadrat* and *Rectangle* route were set up using the FragMAP system (Yi, 2017) (**Figure 2**). Given that fractional vegetation cover (FVC) is one of the key controlling factors in transpiration, photosynthesis and other terrestrial processes, and meanwhile, it is also an important variable that could be used to describe vegetation quality and reflect changes of ecosystem (Graetz et al., 1988; Hirano et al., 2004). In this study, we selected FVC as the *Condition* index, and *Rectangle* routes were set to collect the FVC of sampling points. In brief, 12 way points were set within an area of 20,000 m^2^ (i.e., 100 m × 200 m), the height was set at 20 m and the UAV took one aerial photograph vertically at each way point (to avoiding disturbance factor, e.g., the aerial photographs taken on the rooftop, road, campsite or river were excluded from this study, and finally 3–5 aerial photographs around each sampling point were selected to estimate the FVC (**Figure S1**). The ground resolution was about 1 cm and the FVC could be calculated by the threshold method, the flight mode and data collection were introduced in Chen et al. (2016) and Yi (2017).

The biomass (regarded as *Vigor* index) was estimated by *Quadrat* mode. Briefly, 28 aerial photographs were taken vertically downward and 8 azimuth angles (2 m height), then the aerial photographs were matched and extracted point cloud information to estimate the biomass (Zhang et al., 2018). The richness (regarded as the *Organization* index) and RRD (regarded as the *Resilience* index) were estimated based on the aerial photographs that were collected by *Belt* mode, i.e., all species were identified visually and recorded within each aerial photograph, and the indexes were then calculated for each sample point (there are 74 plant species and 3 ones were not identified on the aerial photographs, see Sun et al., 2018 for details). DJI Mavic Pro UAV was used to take aerial photographs (DJI Innovation Company, China), which equipped with a built-in 20-megapixel RGB camera, and featured with terrain following function.

### 2.3 Calculation of CVOR

The integrated index CVOR (both field- and UAV-based methods, field *_CVOR_* and UAV*_CVOR_*) were calculated followed the previous studies (Hou et al., 2004; Shan et al., 2012; Yu et al., 2018):


CVOR=C×VOR,

where C is the integrated environmental index, C ∈ [0, 1] and it will be valued as 1 if C > 1; VOR is the integrated biological index, VOR = (W*_V_* × V + W*_O_* × O + W*_R_* × R), where W*_V_*, W*_O_* and W*_R_* are the weighting coefficients that indicate the importance of each single index, avoiding the errors that could be caused by spatial heterogeneity and temporal variability. Given that the sampling background was consistent in this study, the values were calculated as W*_V_* = W*_O_* = W*_R_* = 1/3, VOR ∈ [0, 1] and it will be valued as 1 if VOR > 1; CVOR ∈ [0, 1] and it will be valued as 1 if CVOR > 1.

For field *_CVOR_*, the soil organic carbon concentration (SOC) was detected by the K_2_Cr_2_O_7_-H_2_SO_4_ oxidation method (Nelson and Sommers, 1996), and the *Condition* index (C*_SOC_*) for SOC was calculated as:


$${\text{C}}_{SOC}{=\text{SOC}}_{x/}{\text{SOC}}_{ck}$$

where SOC*_x_* and SOC*_ck_* are the concentrations of soil sampled in the sampling points and control plot, respectively.

The aboveground biomass was clipped from the quadrats and oven-dried to constant weight at 65 °C, then the *Vigor* index (V*_fB_*) for aboveground biomass was calculated as:


$${\text{V}}_{fB}={\text{ B}}_{x}/{\text{B}}_{ck}$$

where B*_x_* and B*_ck_* are the aboveground biomass sampled in the sampling points and control plot, respectively.

Different plant functional types’ relative abundance could be regarded as an important factor that affects ecosystem responses to herbivores grazing (de Soyza et al., 2000a; Lunt et al., 2007). The richness was measured by counting all the species within the quadrats, then the *Organization* index (O*_fR_*) for richness was calculated as:


$${\text{O}}_{fR}={\text{ R}}_{x}/{\text{R}}_{ck}$$

where R*_x_ *and R*_ck_* are the richness within quadrats sampled in the sampling points and control plot, respectively.

The RRD was measured by counting the functional groups within the quadrats, then the *Resilience* index (R*_fD_*) for RRD was calculated as:


$${\text{R}}_{fD}=\;\left({\text{P}}_{g}/{\text{P}}_{gck}\right)/\left({\text{P}}_{d}/{\text{P}}_{dck}\right)$$

where P*_g_* and P*_d_* are the proportion of restored and degraded species in the sampling points, and P*_gck_ *and P*_dck_ *are the proportion of restoration and degraded species in control plot, respectively.

For UAV*_CVOR_*, the FVC was retrieved by a threshold method which is conducted a Java-based software (Chen et al., 2016; Yi, 2017), and the *Condition* index (C*_FVC_*) for the FVC was calculated as:


$${\text{C}}_{FVC}={\text{ FVC}}_{x/}{\text{FVC}}_{ck}$$

where FVC*_x_* and FVC*_ck_* are the vegetation coverage in the sampling points and control plot, respectively.

The biomass was measured based on the point cloud from the aerial photographs, and the *Vigor* index (V*_UB_*) for the UAV-based biomass was calculated as:


$${\text{V}}_{UB}={\text{ B}}_{x}/{\text{B}}_{ck}$$

where B*_x_* and B*_ck_* are the aboveground biomass sampled in the sampling points and control plot, respectively.

The richness was measured based on species frequencies occurred in the aerial photographs collected along *Belt* routes, and the *Organization* index (O*_UR_*) for the UAV-based richness was calculated as:


$${\text{O}}_{UR}={\text{ R}}_{x}/{\text{R}}_{ck}$$

where R*_x_* and R*_ck_* are the species frequencies occurred within *Belt* routes sampled in the sampling points and control plot, respectively.

The RRD was measured by counting the functional groups within the *Belt* routes, and the *Resilience* index (R*_UD_*) for the UAV-based richness was calculated as:


$${\text{R}}_{UD}=\;\left({\text{P}}_{g}/{\text{P}}_{gck}\right)/\left({\text{P}}_{d}/{\text{P}}_{dck}\right)$$

where P*_g_* and P*_d_* are the proportion of restoration and degraded species in sampling points, and P*_gck_ *and P*_dck_ *are the proportion of restoration and degraded species in control plot, respectively.

### 2.4 Data analysis

To test the normality of data, a goodness-of-fit test (based on Shapiro-Wilk test and univariate procedure) was used firstly for all the collected data. For the regression analysis, the R^2^ and *P* values were used to compare the performance of sampling methods (i.e., UAV*_CVOR_* vs. field *_CVOR_*). To select the final regression models which indicated the effects of grazing intensity on GHA, the likelihood ratio tests were used to compare the regression models (n = 27), and select the optimal regression models to reveal the relationships between grazing intensity and GHA. The statistical analyses were performed with R version 4.1.3.

## 3 Results

### 3.1 The characteristics of UAV*_CVOR_*

**Figure 3:**
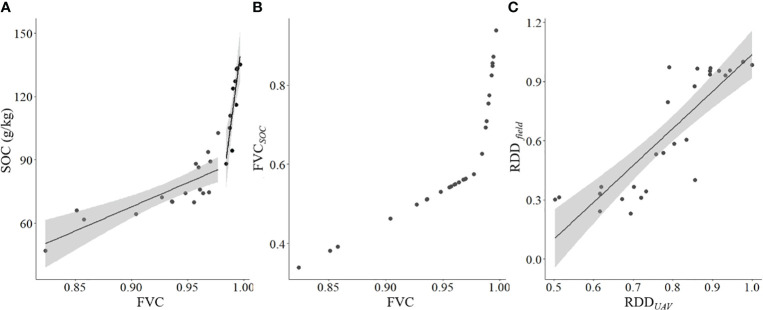
Relationships among indicators that estimated by field- and UAV-based methods. **(A)** relationship between vegetation coverage (FVC) and soil organic carbon concentration (SOC) (SOC = 228.29 FVC -137.71, R^2 =^ 0.612, *P*< 0.001 when FVC< 0.980, and SOC = 3938.6 FVC - 3786.9, R^2 =^ 0.725, *P*< 0.001 when FVC ≥ 0.980); **(B)** relationship between the transitional indicator (FVC*_SOC_*) and actual measured FVC, **(C)** relationship between ratio of the species that indicate restoration and degraded degree (RDD) that estimated by field- and UAV-based methods (RRD*_field_* = 1.87 RRD*_UAV_* - 0.84, R^2 =^ 0.713, *P*< 0.001).

The SOC increased significantly with the increase of FVC, but the rate of increase was significantly faster when FVC≥ 0.98 compared to that when FVC< 0.98, showing a segmented function between the SOC and FVC (**Figure 3A**). After transformation based on the segmented function, the FVC was positively corelated to the transitional indicator (FVC*_SOC_*, **Figure 3B**), reflecting the condition of the sample points. RRD could be extracted from aerial photographs that were collected by FragMAP system and analyzed easily and efficiently, and it was significant corelated to the field-based RRD (**Figure 3C**).

**Figure 4:**
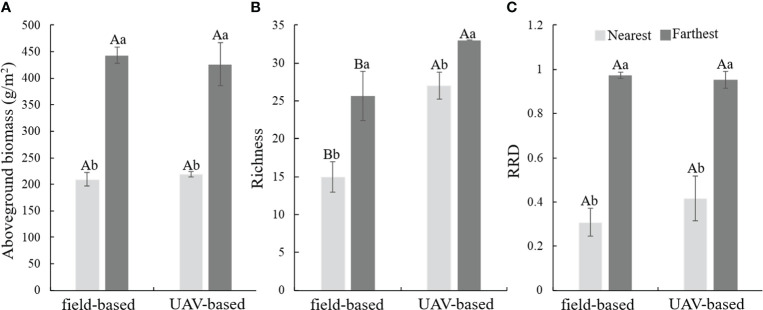
Comparation of the biological index of the nearest and farthest sample points of the tree household pastures that estimated by field- and UAV-based method. **(A)** aboveground biomass, **(B)** richness, **(C)** ratio of the species that indicate restoration and degraded degree (RRD).

Compare to the nearest sample sites from campsites, the farthest sample sites exhibited higher estimated values for aboveground biomass, richness and RDD (**Figure 4**, *P*< 0.05). The UAV-based method estimated higher values of the richness both at the nearest and farthest sample points, and the difference was greater at the nearest sample points (**Figure 4B**).

### 3.2 Comparation of the CVORs that were assessed by field- and UAV-based methods

**Figure 5:**
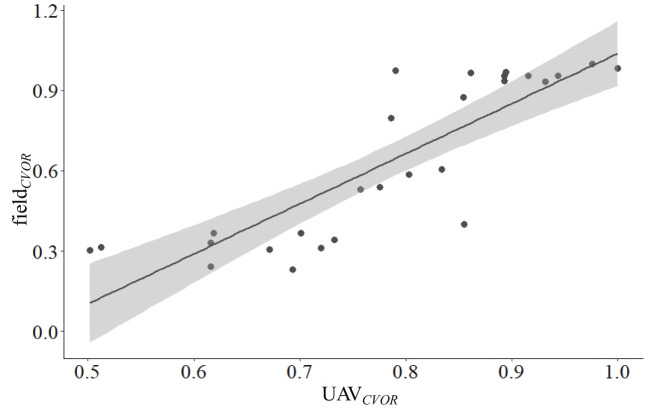
Relationships between grassland health assessment integrated index that were estimated by the traditional field- and UAV-based methods (field*_CVOR_* = 1.87 UAV*_CVOR_* - 0.84, R^2 =^ 0.713, *P*< 0.001).

There were significant linear relationships between CVOR estimated by the field- and UAV-based methods (*P*< 0.001, **Figure 5**). The variation range (0.44 – 0.98) of values estimated by UAV-based method was smaller than that (0.15 – 0.93) estimated by the field-based method, especially around areas with higher grazing intensities.

### 3.3 Relationships between GHA and grazing intensity

**Figure 6:**
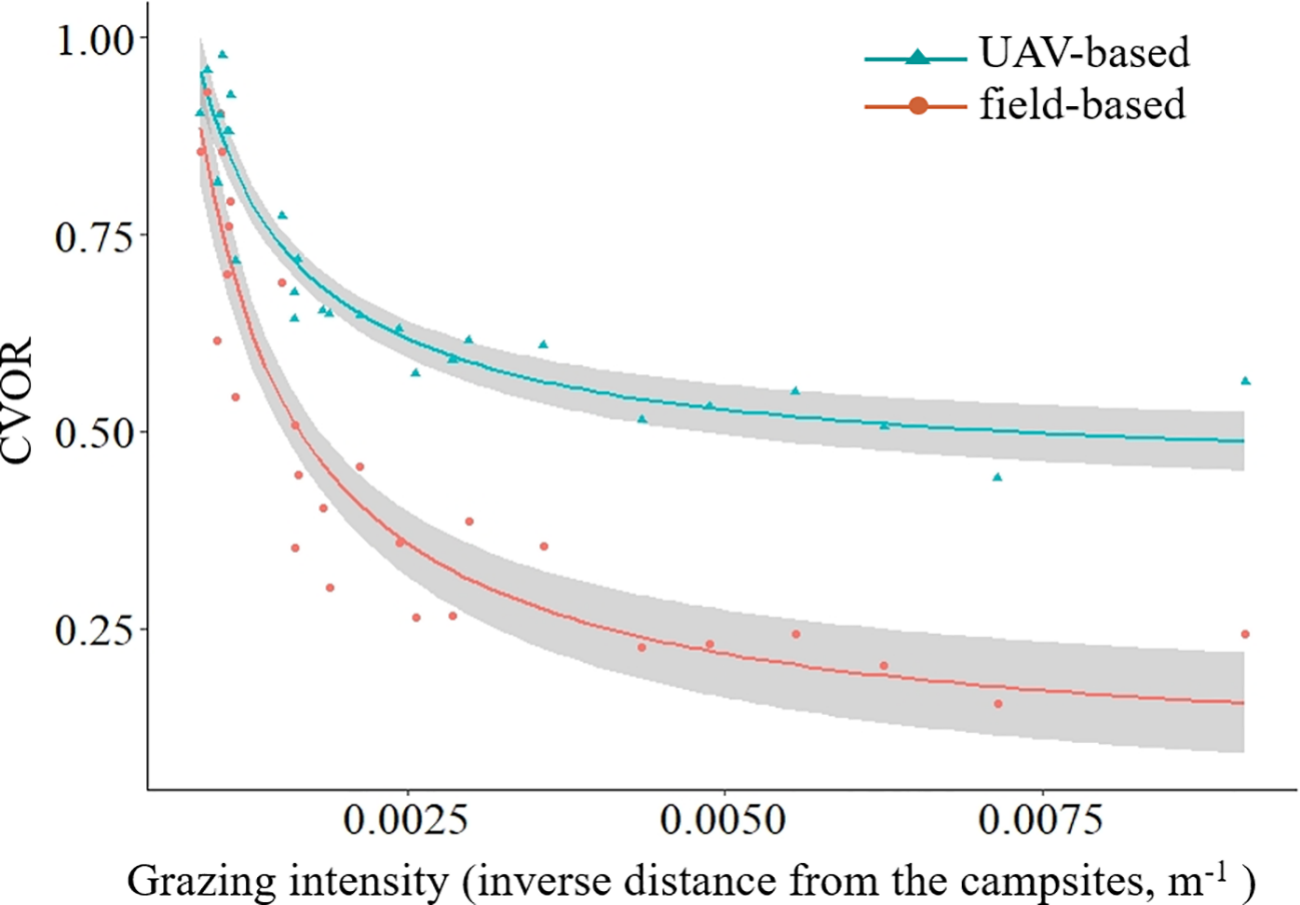
The grassland health assessment integrated indicator (COVR) along the grazing intensity (field*_CVOR_* = 0.0000268 GI ^-1.45 +^ 0.176, R^2 =^ 0.866, P< 0.001; UAV*_CVOR_* = 0.0000926 GI ^-1.22 +^ 0.474, R^2 =^ 0.884, P< 0.001).

CVOR index was initially decreased nonlinearly, and then kept stable with increasing grazing intensity (**Figure 6**). Although the tendency was same, the UAV*_CVOR_* exhibited higher values than that estimated by the field-based method for the same sampling point, and furthermore, the differences increased with the increasing grazing intensity (**Figure 6**).

## 4 Discussion

### 4.1 UAV*_CVOR_* establishment for GHA

It is challenged for grassland managers to make management decisions across large landscapes based on limited time and financial resource (Lepak et al., 2022). GHA is a bridge of grassland study and management (Xu and Guo, 2015). Followed the methodology of Hou et al. (2004); Yu et al. (2018) and etc., we established the integrated index-CVOR based on the indicators represent the environmental conditions and biological characteristics (i.e., C + VOR). Similar to the SOC concentration, the vegetation coverage is regarded as a sensitive indicator of land degradation and desertification (Hirano et al., 2004; Jiapaer and Bao, 2011). In this study, the relationship between FVC and SOC was a segmented function (**Figure 5**), but the normalized transformation made it possible to indicate the SOC level by FVC*_SOC_*. In addition, in this study, the vegetation and soil type were similar, thus the FVC could be an appropriate indicator for the *Condition* index.

Regarding the biological indexes, it has been found that the UAV-based method could estimate the aboveground biomass and richness of alpine grassland efficiently and correctly (Sun et al., 2018; Zhang et al., 2018). Similarly, the UAV-based method could also be used to estimate the RRD (the significant linear relationship between the values that measured by UAV- and field-based methods, **Figure 3C**). Therefore, the CVOR estimated by UAV-based method is reliable and efficient.

### 4.2 Changes of GHA along grazing intensities

Quantitative data describing condition gradients of specific ecosystem will be of utility in assessment, monitoring, and sustainable management of grassland ecosystems (Bosch and Kellner, 1991; Wei et al., 2022b), meanwhile, it is also an efficient study method to the scientists engaging in grassland ecosystem related research activities (Herrick et al., 2006). Specifically, resource inhomogeneous distribution or management mode could result in phenomena of radial gradient of grazing intensity in specific areas such as household pastures, which is regarded as a useful way to understand and predict the relationships between grasslands and herbivores (Chillo et al., 2015; Sun et al., 2018). The superior advantage is that it includes multi-grazing intensities, rather than the only grazing & enclosure or several specific grazing intensities treatments (Lunt et al., 2007), which is helpful to reveal the potential relationships between grazing intensity and the response indictors (Sun et al., 2018; Teague and Kreuter, 2020). In this study, we demonstrate a nonlinear relationship between the CVOR (estimated by field- and UAV-based methods) and grazing intensities (**Figure 6**). Meanwhile, compared to traditional field-based method, the UAV-based method could sample within larger ranges both for single monitor unit and total monitor areas, which meaning the higher representativeness and lower errors for the specific monitor area (Sun et al., 2018), especially for the species richness (**Figure 4B**). Furthermore, the higher representativeness improves estimation accuracy (Fuhlendorf and Engle, 2001), especially for the grassland ecosystem featured with heterogeneity. In this study, the CVOR values estimated by the UAV-based method were higher than those measured by the field-based method could be resulted from the higher representativeness (**Figures 5** and 6, Sun et al., 2018). Hence, we conclude that the UAV-based method is appropriate for GHA, and it could be an optional way for scientific research and practical guidance, such as provides dynamic optimum grazing intensities at various scales according to the GHA, and fills the gap between botanical characteristics that were studied in plot experimental and natural communities (Genung et al., 2020; Sun et al., 2022).

### 4.3 Advantages of UAV-based GHA

In general, different biophysical parameters of grasslands require scale-matched monitoring method prior to capture their spatial or temporal variation (Bradley and Millington, 2006; He et al., 2006; He et al., 2007). Thus, it is better if the methodology of GHA could include analyses at multiple spatio-temporal scales. However, up to now, it is still a challenge to find suitable scale (both temporal and spatial scales) thresholds for GHA modeling (Xu and Guo, 2015). In recent years, the UAVs had been used to fill the spatial gap between the field-based sampling and satellite images (Chen et al., 2016; Zhang et al., 2023), which overcomes the scale issue for ecological studies. Similarly, the UAVs controlled by FragMAP system could be used for GHA estimate within a similar phenological period at a large scale. The results would be more precise because of the larger sampling area and higher representativeness, and which could reduce sampling bias in the heterogeneous landscapes (Sun et al., 2018). Besides, the UAVs can reach the areas where are difficult for human walking (e.g., wetland), and data are collected efficiently (Floreano and Wood, 2015). Therefore, the UAV-based method makes it possible to carry out GHA *in situ* at a large scale, which is especially useful on the QTP featured with high elevation and low oxygen (Sun et al., 2022).

Generally, GHA requires long-term repeated monitoring to realize comparability (Arif et al., 2016; Lepak et al., 2022). The UAV-based method (controlled by FragMAP system) proposed in this study realized flights repeatedly and efficiently at different times with fixed height (Yi, 2017; Sun et al., 2018). Meanwhile, the large numbers of sampling locations that represent general patterns of GHA among ecological sites (de Soyza et al., 2000a). Moreover, the UAV-based noninvasive sample method could keep the sample sites at ideal state which makes sure that the results are more believable and comparable, and it is crucial for the control or reference sites that could be destroyed by sampling repeatedly (Lepak et al., 2022).

### 4.4 Limitations of UAV*_CVOR_* and suggestions to improve UAV-based GHA

We proposed and tested the UAV-based GHA method at typical household pastures on the QTP. The method exhibited more efficiency and representativeness of larger sample aeras (e.g., Sun et al., 2018). However, we do acknowledge the limitations of UAV-based GHA method. First, some species such as the creeping or low-growing plants in UAV photographs could not be identified (Sun et al., 2018). Fortunately, higher resolution of UAVs could allow us to identify detail information, meanwhile, taking additional aerial photographs manually at close distance (e.g., 0.5 m) will make it possible to find detail under the canopy as the airflow made by the UAVs’ propellors (Sun et al., 2018). Second, to date, the visual identification process requires substantial time and professional knowledge, the target identification automatically by increasing the machine learning algorithm could further promote the efficiency of UAV-based method (Lu and He, 2017; Menshchikov et al., 2021).

Though we demonstrated the feasibility of UAV*_CVOR_* at the household pastures in this study, the feasibility of the UAV-based integrated indexes to different types of grassland at multiple scales is still necessary. Meanwhile, the specific indicators included in CVOR could make adjustments according to study need, e.g., taking the bioclimatic dataset (download from www.worldclim.org/current or other datasets) as *Condition* index at regional scale. In addition, with filling special gap between the traditional field-based samples and satellite images by UAV technology, the standardized space-air-ground integrated observation system for GHA could be established, and it will be easier to select the control or reference sites scientifically around the large areas. Finally, we may integrate the single monitor points and thus improve the datasets, which will be beneficial to vertical and horizontal comparison and applied in site-specific management strategies and environmental protection (Miller, 2008; Ye et al., 2011).

It is usually hard to separate the effects caused by grazing and succession in ecosystems featured with the dynamic succession processes. Thus, the long-term fixed points monitor system is urgent to develop to solve the confusing issues. The UAV-based sampling processes of GHA was separated into two parts: cooperative UAV-field sampling and net-cooperation data extraction (Sun et al., 2022). UAV-based sampling is more efficiency which could reduce the field work time, and net-cooperation data analysis could be carried out synergistically in laboratories, which overcome the temporal and spatial limitations of data analysis of GHA (e.g., the species composition, Sun et al., 2018); and more important is that the datasets dased on aerial photographs could be the critical point in integrating the qualitative or quantitative protocols (Lepak et al., 2022). Therefore, the UAV*_CVOR_* will contribute to study real-world conditions at a large scale, which could be helpful to separate the effects caused by disturbance and succession, and will contribute to the sustainable development of grassland ecosystem.

## Data availability statement

The original contributions presented in the study are included in the article/**Supplementary Material**. Further inquiries can be directed to the corresponding author.

## Author contributions

Conceptualization, SY and YS; methodology, YS, QM, and SY; software, YS and YLuo; validation, YY, GD, QM, and YLuo; investigation, YLiao, WJ, WW and YS; data curation, YS, YY and XW; writing-original draft preparation, YS, YLuo, WJ, WW and XW; writing-review and editing, SY and YS; visualization, YLiao, GD, and YY; supervision, SY; project administration, YS. All authors contributed to the article and approved the submitted version.
