# Supplementary material for: Grassland health assessment based on indicators monitored by UAVs: a case study at a household scale
Source: Front Plant Sci. 2023 Sep 20;14:1150859. doi: 10.3389/fpls.2023.1150859 (PMC10548208; doi:10.3389/fpls.2023.1150859)
Supplement: Supplementary file 1 [file DataSheet_1.docx]

***Supporting information***


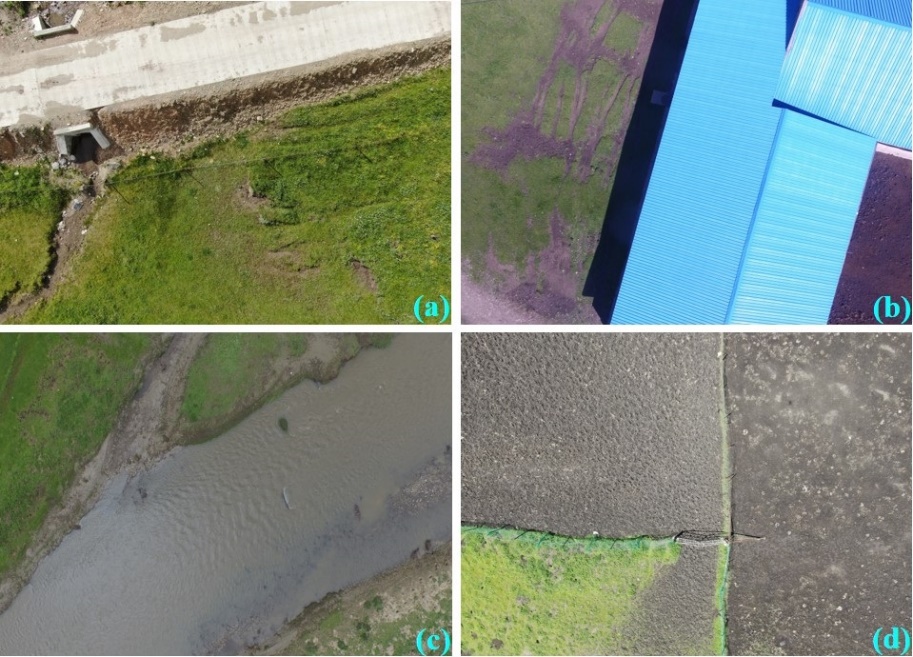


**Figure S1** The aerial photographs contain (a) road, (b) rooftop, (c) river and (d) campsite.
